# Author Correction: Coexisting ecotypes in long-term evolution emerged from interacting trade-offs

**DOI:** 10.1038/s41467-023-43839-2

**Published:** 2023-12-05

**Authors:** Avik Mukherjee, Jade Ealy, Yanqing Huang, Nina Catherine Benites, Mark Polk, Markus Basan

**Affiliations:** Harvard Medical School, Department of Systems biology, 200 Longwood Avenue, Boston, MA 02115 USA

**Keywords:** Evolutionary theory, Microbial ecology, Experimental evolution

Correction to: *Nature Communications* 10.1038/s41467-023-39471-9, published online 26 June 2023

In this article, the funding from ‘the Systems, Synthetic and Quantitative Biology PhD program training award T32GM135014’ was omitted. The original article has been corrected.

