# Supplementary material for: Coexisting ecotypes in long-term evolution emerged from interacting trade-offs
Source: Nat Commun. 2023 Jun 26;14:3805. doi: 10.1038/s41467-023-39471-9 (PMC10293278; doi:10.1038/s41467-023-39471-9)
Supplement: Supplementary file 1 — Supplementary Information [file 41467_2023_39471_MOESM1_ESM.pdf]

Coexisting ecotypes in long-term evolution emerged from competing trade-offs

Mukherjee et al.

Table of Contents

Mathematical model and computer simulation 2
Model Initialization 2
Daily dilution 2
Growth on glucose 2
Acetate excretion trade-off 2
Glucose consumption 3
Lag time to acetate 3
Acetate consumption 4
Model parameters 4
Simulation 5
Supplementary Figures 6
Supplementary References 11

Mathematical model and computer simulation

Model Initialization

The experiment is initialized with an initial glucose concentration $[Glu] = [Glu]_0$, based on the concentrations used in the LTEE[1], and an initial acetate concentration $[Ace] = [Ace]_0$, which is typically zero. The abundances each one of N total strains are reflected in their optical densities, denoted by OD_i.

Daily dilution

After each 24-hour interval in simulation time, optical densities were rescaled by the dilution factor, $f = 1/100$, chosen based on the LTEE protocol[1]. Substrate concentrations were reset to the initial concentrations $[Glu] = [Glu]_0$ and $[Ace] = [Ace]_0$.

Growth on glucose

We denote all metabolite concentrations in units of carbon atoms to simplify conversion between metabolites. We assume that the strain preferentially utilize glucose irrespective of the acetate concentration and only switch to acetate once glucose is depleted. Therefore, while glucose concentration $[Glu] > 0$, irrespective of the acetate concentration $[Ace]$, we assume that the optical densities OD_i, grow at their respective constant glucose growth rates, denoted λ_i^G:

$$\frac{dOD_i}{dt} = \lambda_i^G OD_i. \qquad \text{[S1]}$$

The strain-specific growth rate on glucose λ_i^G is the only parameter that we vary between strains.

Acetate excretion trade-off

We used the parameters for the two Pareto fronts given by Eqs. [1-2] in the main text that were determined for the NCM3722 strain in previous studies [2,3]. To speed up the simulation, rather than the inequalities given by Eqs. [1-2], we assumed that phenotypes are directly on the Pareto fronts, as a function of only their glucose growth rates λ_i^G. Hence, acetate excretion rates of each strain are given by

$$j_i^{ace} = \alpha(\lambda_i^G - \lambda_0)\theta(\lambda_i^G - \lambda_0), \qquad \text{[S2]}$$

where θ is the Heaviside step function and the proportionality constant α and the threshold growth rate λ_0 have been empirically determined previously for the strain NCM3722 [2].

During growth on glucose $[Glu] > 0$, the concentration of acetate $[Ace]$ evolved in the simulation, according to acetate excretion rates of each strain j_i^{ace}, given by Eq. [S2] and the abundances of each strain OD_i:

$$\frac{d[Ace]}{dt} = \sum_{i=1}^{N} j_i^{ace}\, OD_i. \qquad \text{[S3]}$$

Glucose consumption

The rate of acetate excretion affects the biomass yield y_i^G of each strain, which is defined as the amount of biomass produced per glucose consumed. The change in glucose due to the growth of a single strain can be written as

$$\frac{d[Glu]}{dt} = -\frac{dOD_i}{dt}/y_i^G = -\lambda_i^G\, OD_i/y_i^G = -\lambda_i^G\, OD_i/y_0^G - j_i^{ace} OD_i, \quad \text{[S4]}$$

where j_i^{ace} is the strain specific acetate excretion rate given by Eq. [S2] and y_0^G is the biomass yield on glucose in the absence of acetate excretion. Note that for simplicity, we neglect the small change in biomass yield that comes from the change in respiration flux that coincides with changes in fermentation flux[2]. Combining Eq. [S4] with Eq. [S2], the growth rate dependent biomass yield on glucose is then given by

$$1/y_i^G = 1/y_0^G + \alpha\left(1 - \lambda_0/\lambda_i^G\right)\theta\left(\lambda_i^G - \lambda_0\right). \quad \text{[S5]}$$

The evolution of the glucose concentration $[Glu]$ during growth on glucose is then given by the sum of glucose consumption of all the strains in the population

$$\frac{d[Glu]}{dt} = -\sum_{i=1}^{N} \lambda_i^G\, OD_i/y_i^G. \quad \text{[S6]}$$

Note that for simplicity, we assume that glucose runs out quasi-instantaneously due to an infinitely small Michaelis constant of glucose uptake.

Lag time to acetate

We assume that strains only shift to acetate utilization once glucose is depleted. However, based on the second trade-off, given by Eq. [2] in the main text, cells cannot immediately switch to growth on acetate, but experiences a lag time instead. Lag time is the time lost in the switch, as compared to instantaneous growth at steady-state growth rate on acetate λ^A, which we assume to be identical for all strains. Because strains that are not on the Pareto front are clearly sub-optimal, we assume that all phenotypes are directly on both Pareto fronts. Therefore, lag time T_i^{lag} is directly given by the expression in Eq. [2] of the main text

$$T_i^{lag} = \beta/\left(\lambda_C - \lambda_i^G\right), \quad \text{[S7]}$$

where β is a proportionality constant and λ_C is the critical growth rate, which is the maximum growth rate achievable and the growth rate where lag times become infinite and the strain is unable to utilize acetate at all. To implement lag phases in the simulation, we set growth rates of each strain to zero for the duration of their respective lag time T_i^{lag}, counting from the time when glucose in the medium has run out, denoted by T^{switch}. The change in optical density after glucose has run out is then given by

$$\frac{dOD_i}{dt} = \lambda^A \theta\left(T^{switch} - T_i^{lag}\right) OD_i, \qquad [S8]$$

where θ is the Heaviside step function.

Acetate consumption

Acetate in the medium is then consumed according to the growth rates in Eq. [S8]

$$\frac{d[Ace]}{dt} = -\sum_{i=1}^{N} \lambda^A \theta\left(T^{switch} - T_i^{lag}\right) OD_i / y^A, \qquad [S9]$$

where y^A is the biomass yield for growth on acetate. Note that for simplicity, we assume that acetate runs out quasi-instantaneously due to an infinitely small Michaelis constant of acetate uptake.

Model parameters

**Description and references**	**Model symbol**	**Value**	**Unit**	**Simulation parameter**
Initial glucose concentration[1]	$[Glu]_0$	0.834*	mM	initialconcG
Initial acetate concentration[1]	$[Ace]_0$	0	mM	initialconcA
Dilution factor[1]	f	1/100	1	f
Acetate threshold growth rate[2]	λ_0	0.76	1/hr	lambda0
Acetate excretion slope[2]	α	20.0*	mM/OD	alpha
Inverse biomass yield on glucose without acetate excretion[2]	$1/y_0^G$	37.0*	mM/OD	sigma
Critical growth rate[3]	λ_C	1.1	1/hr	lambdaC
Lag coefficient[3]	β	0.7	1	beta
Maximum growth rate on acetate[3]	λ^A	0.5	1/hr	lambdaA(i)
Inverse biomass yield on acetate	y^A	37.0*	mM/OD	yA(i)

* in units of carbon atoms

Simulation

The model was implemented in MATLAB and the dynamic equations were integrated using a discrete timestep. We checked that the timestep was sufficiently small, such that the dynamics were independent of the timestep.

Supplementary Figures

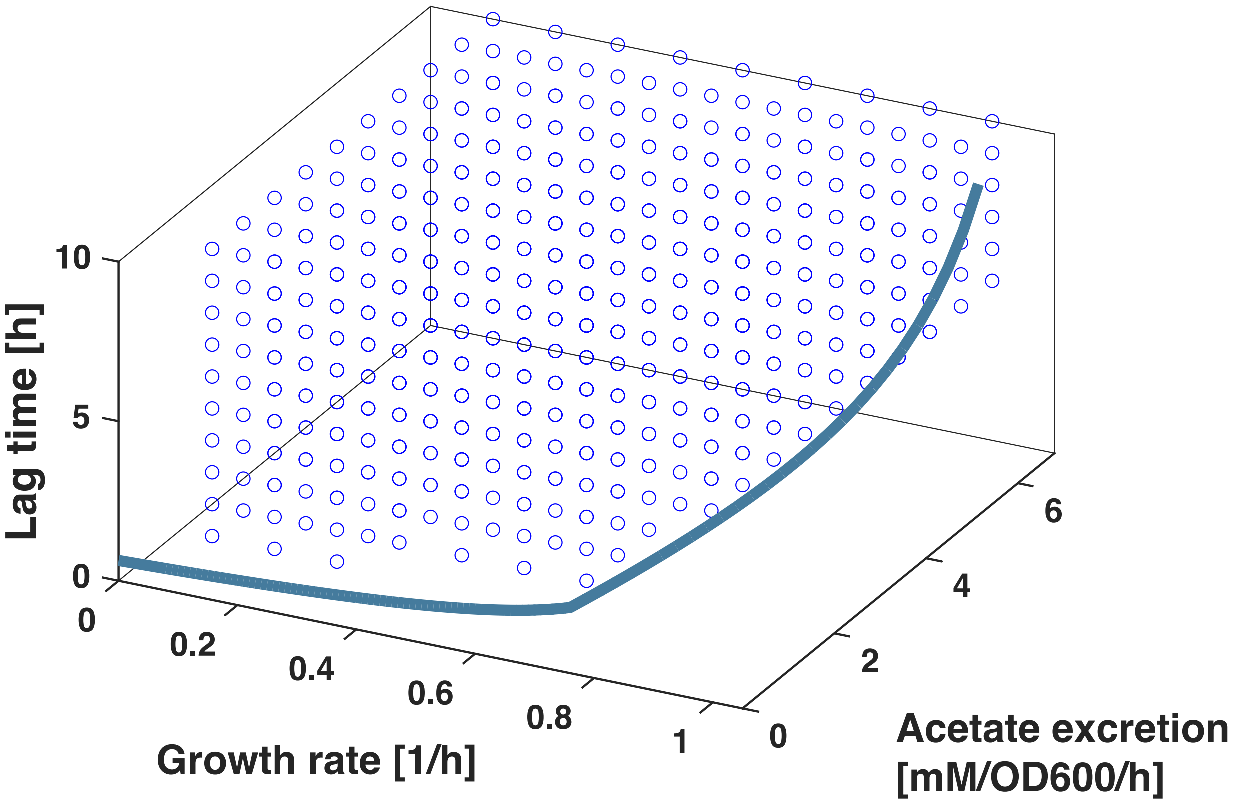

Fig. S1: Accessible phenotypic space from combination of two trade-offs. *The trade-offs given by Eqs. [1-2] of the main text are combined in this 3D plot of growth rate versus acetate excretion rate versus lag time. The region of accessible phenotypes is filled by the blue markers. The solid line is the curve of 'optimal' phenotypes that are directly on the intersection of the two Pareto fronts, and thus satisfy Eqs. [1-2] as exact equalities.*

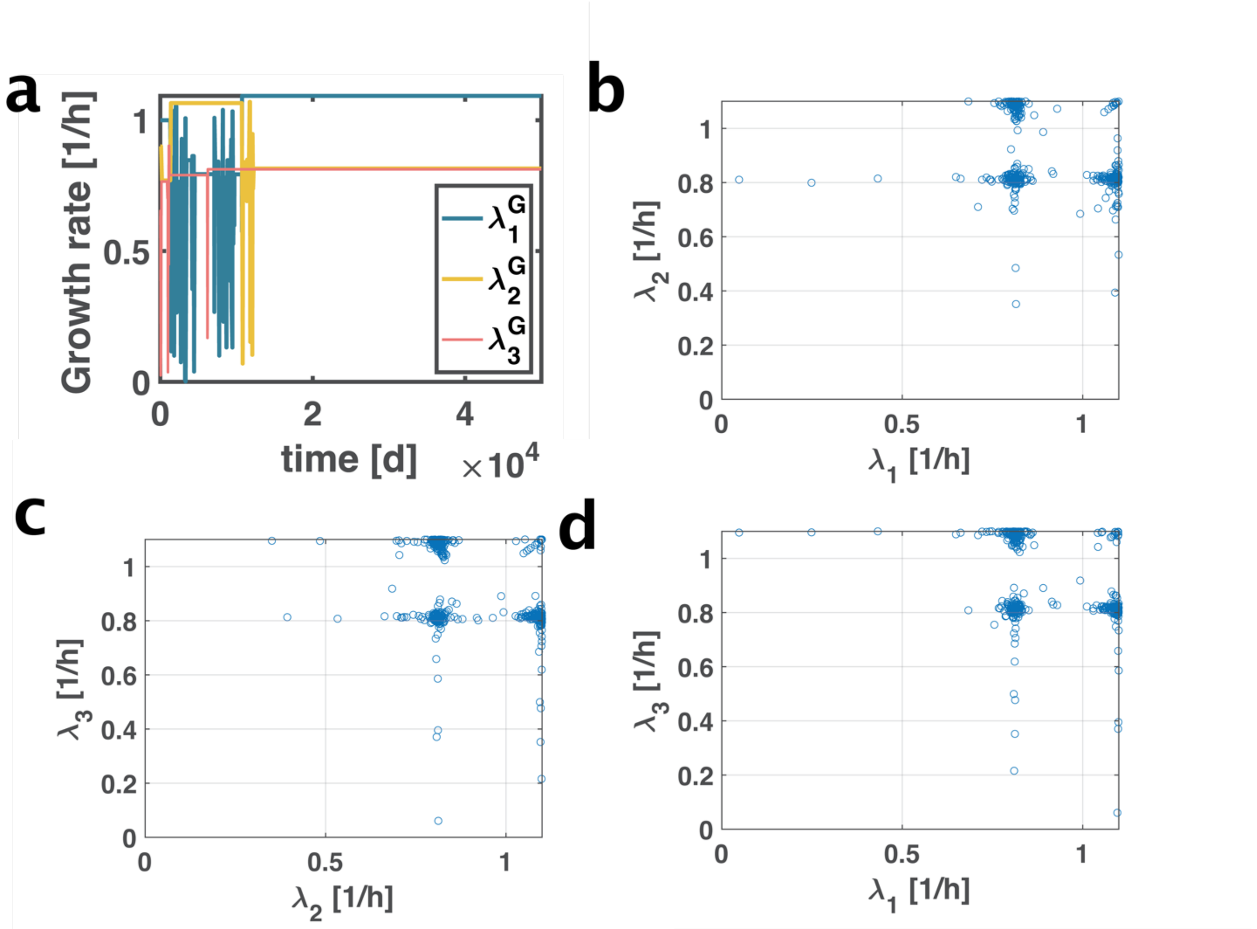

Fig. S2: Convergent evolutionary dynamics. a, *Growth rates of three strains in the simulation as a function of time. Every time the abundance of one of the three strains drops below a low threshold level indicating elimination, a new strain with a randomly chosen growth rate is introduced. The convergence to a specific combination of strains observed in the evolutionary dynamics in Fig. 2b in the main text was further tested.* ***b,c,d,*** *2D projections of Fig. 2e of the main text. Each data point is a combination of three final growth rates of one evolutionary simulation after 50,000 days of simulated evolution. The projections show clusters at different combinations of the two fixed-point growth rates. In the two 2D projections, there are also clusters at two identical growth rates. However, the 3D plot shown in Fig. 2e of the main text reveals that there are no clusters at three identical growth rates (red spheres) and the third growth rate of these clusters that is not visible in the 2D projection is the other fixed-point growth rate.*

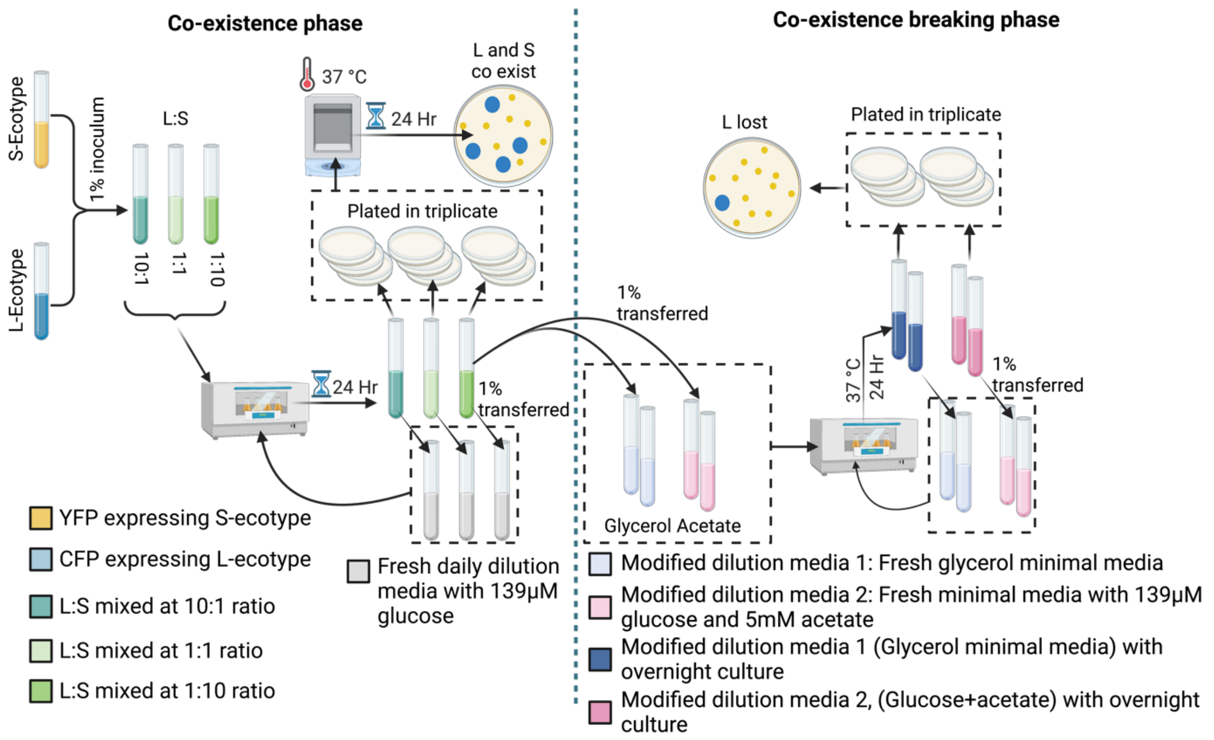

Fig. S3: Illustration of experimental design of the daily dilution experiment. *L ecotype (blue) and S ecotype (yellow) were separately grown overnight in N+C+ minimal medium with typical glucose concentration of the LTEE (139µM) (daily dilution medium). The next morning fresh daily dilution medium was inoculated with L- and S-strain from overnight culture in different ratios (L:S= 10:1and 1:1 and 1:10). Co-culture tubes in different ratios (shades of green) were grown at 37° C in a shaker incubator with 200 RPM orbital shaking. Every 24 hours, cultures were transferred to tubes containing fresh daily dilution medium with a 1:100 dilution and continued to incubate. Bacterial samples for plating were taken before dilution from overnight saturated culture and plated in triplicate. Plates were incubated overnight and the next morning colonies were imaged in a custom-built setup. Upon reaching stable coexistence, the culture tube was split into three cultures with two types of modified media and the same daily dilution medium as a control. The first modified medium contained a large amount of acetate (5 mM) in addition to normal amount of glucose (139µM). For the second modified medium, glucose was replaced with equivalent amount of glycerol.*

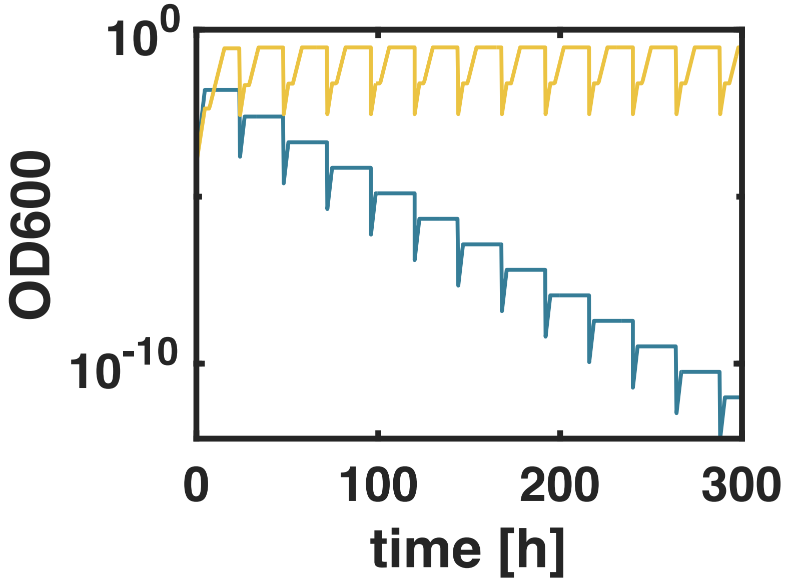

Fig. S4: Daily dilution simulation with normal glucose (139µM) and high acetate in the medium (5 mM). *Coexistence disappears and the faster-growing L-strain on glucose is eliminated, because the slower growing S-strain no longer benefits from the higher acetate excretion rate of the L-strain.*

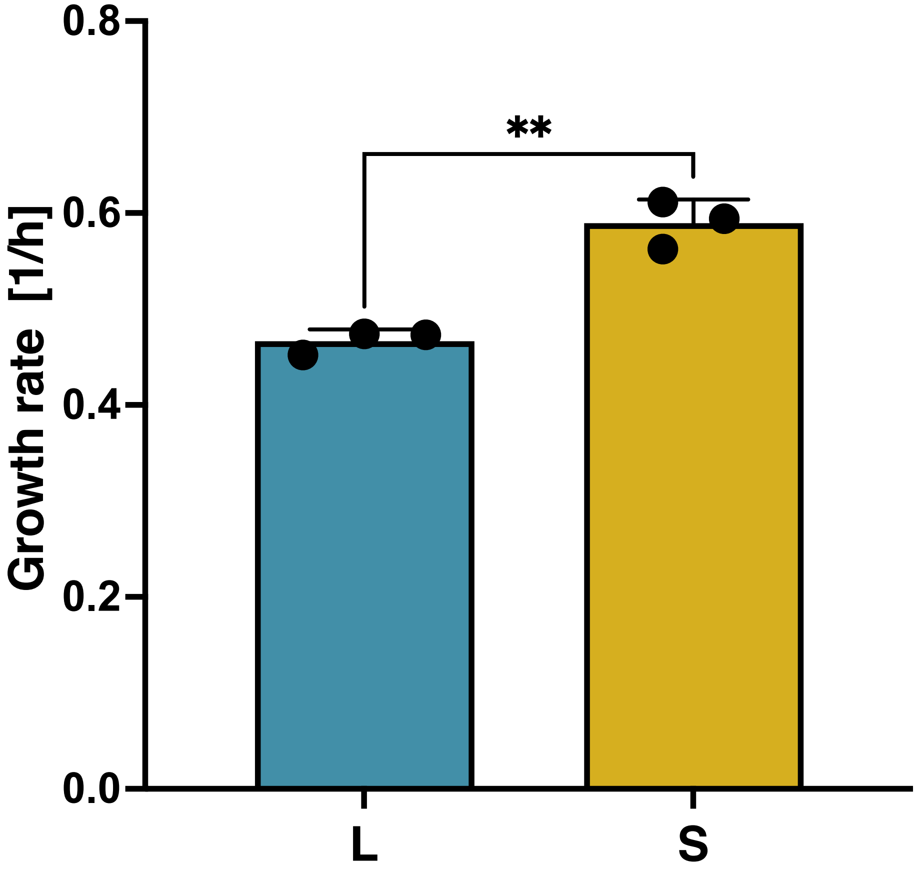

Fig. S5: Steady-state growth rate of L and S in N+C+ glycerol minimal medium. *L and S strains were grown (n=3, biological replicates) in batch culture in N+C+ minimal medium supplemented with 20 mM* NH_4Cl *and 40 mM glycerol. L strain has a mean growth rate 0.47±0.01* h^{-1} *and S strain has a mean growth rate 0.59±0.03* h^{-1}*. Error bars represent standard deviation (unpaired t-test, P value=0.0015).*

Supplementary References

1. Good, B. H., McDonald, M. J., Barrick, J. E., Lenski, R. E. & Desai, M. M. The dynamics of molecular evolution over 60,000 generations. *Nature 2017 551:7678* **551**, 45–50 (2017).
2. Basan, M. *et al.* Overflow metabolism in Escherichia coli results from efficient proteome allocation. *Nature* **528**, 99–104 (2015).
3. Basan, M. *et al.* A universal trade-off between growth and lag in fluctuating environments. *Nature* (2020) doi:10.1038/s41586-020-2505-4.
